# Cyanobacterial Toxic and Bioactive Peptides in Freshwater Bodies of Greece: Concentrations, Occurrence Patterns, and Implications for Human Health

**DOI:** 10.3390/md13106319

**Published:** 2015-10-12

**Authors:** Spyros Gkelis, Thomas Lanaras, Kaarina Sivonen

**Affiliations:** 1 Department of Botany, School of Biology, Aristotle University of Thessaloniki, GR-541 24 Thessaloniki, Greece; E-Mail: lanaras@bio.auth.gr; 2 Department of Food and Environmental Sciences, Division of Microbiology and Biotechnology, University of Helsinki, P.O. Box 56, FI-00014 Helsinki, Finland; E-Mail: kaarina.sivonen@helsinki.fi

**Keywords:** microcystins, anabaenopeptins, anabaenopeptilides, cyanobacteria, harmful algal blooms, Greece, risk assessment

## Abstract

Cyanobacterial harmful algal blooms represent one of the most conspicuous waterborne microbial hazards in aquatic environments mostly due to the production of toxic secondary metabolites, mainly microcystins (MCs). Other bioactive peptides are frequently found in cyanobacterial blooms, yet their concentration and ecological relevance is still unknown. In this paper we studied the presence and concentration of cyanobacterial peptides (microcystins, anabaenopeptins, anabaenopeptilides) in 36 Greek freshwater bodies, using HPLC-DAD, ELISA, and PP1IA. Microcystins were found in more than 90% of the samples investigated, indicating that microcystin-producing strains seem to also occur in lakes without blooms. Microcystins MC-RR, MC-LR, and MC-YR were the main toxin constituents of the bloom samples. Anabaenopeptin A and B were predominant in some samples, whereas anabaenopeptolide 90A was the only peptide found in Lake Mikri Prespa. The intracellular concentrations of anabaenopeptins produced by cyanobacterial bloom populations are determined for the first time in this study; the high (>1000 µg·L^−1^) anabaenopeptin concentration found indicates there may be some impacts, at least on the ecology and the food web structure of the aquatic ecosystems. The maximum intracellular MC values measured in Lakes Kastoria and Pamvotis, exceeding 10,000 µg·L^−1^, are among the highest reported.

## 1. Introduction

Cyanobacterial Harmful Algal Blooms (or CyanoHABs) represent one of the most conspicuous waterborne microbial hazards to human and agricultural water supplies, fisheries production, and freshwater and marine ecosystems [1]. This hazard results from the production of cyanotoxins, harmful secondary metabolites such as microcystins (MCs), saxitoxins, and cylindrospermopsins that can have deleterious effects within reservoirs and in downstream receiving water systems during releases [2]. The most frequent cyanotoxins in freshwater CyanoHABs are MCs, cyanobacterial heptapeptide hepatotoxins produced by cyanobacterial species belonging to *Microcystis*, *Planktothrix*, *Anabaena* (*Dolichospermum*), and *Anabaenopsis* (for a review see [2,3]). MCs are responsible for, by several exposure routes, a significant health hazard to livestock, wildlife, birds, fishes, and even humans [4], leading the World Health Organization (WHO) to propose implementation measures for monitoring and control of MCs and determine guideline values for drinking [5] and recreational [6] waters.

During the past 20 years, many other bioactive peptide groups have been discovered in cyanobacteria, such as aeruginosins, microginins, cyanopeptolins, anabaenopeptins, anabaenopeptilides, microviridins, and nostophycins; to date, more than 600 cyanobacterial peptides have been described (for a review, see [7]). The continuous and rising interest stems both from the surveillance of aquatic systems, especially where toxic compounds are raising concerns of public health, and from various and diverse bioactivities of unique structures with potential pharmacological implications [7]. These peptides are frequently found in cyanobacterial blooms, along with numerous other not-yet-identified peptides [8,9,10]. Anabaenopeptins are unique cyclic peptides that have the common cyclic peptide moiety linked with Tyr, Arg, Lys, and Phe, via a ureido bond [11,12]. Anabaenopeptilides are 19-membered cyclic depsipeptides containing a unique residue, 3-amino-6-hydroxy-2-piperidone (Ahp) bond [11,12]. These peptides exhibit diverse bioactivities such as serine, trypsin, or chymotrypsin protease inhibition [7,10]. A better understanding of the production and toxicity of these peptides is essential to assess the environmental controls on peptide production and to guide cyanotoxin risk assessment [13]. Analyses of several toxic and non-toxic strains of cyanobacteria have shown that cyanobacteria may produce MCs and/or other peptides [13,14,15]. Therefore, a comprehensive understanding of the possible functions of bioactive peptides and their ecological impacts requires considering bioactive peptides as a group rather than focusing solely on MCs.

The presence of MCs [16,17,18,19] and anabaenopeptins [16,20] has already been documented in *Microcystis*- and/or *Anabaena* (*Dolichospermum*)-dominated blooms, in Greece. The warm Mediterranean climate favors cyanobacteria blooms in eutrophic waters, which may start in spring and last until December [21], with MCs present throughout the year [17]. In this work we performed extensive sampling along many continental Greek lakes and reservoirs of different typology and trophic status, in order to further investigate the presence and concentration of cyanobacteria peptides (microcystins, anabaenopeptins, anabaenopeptilides) in the water and assess the possible risks posed.

## 2. Results

### 2.1. Peptide Diversity

**Figure 1:**
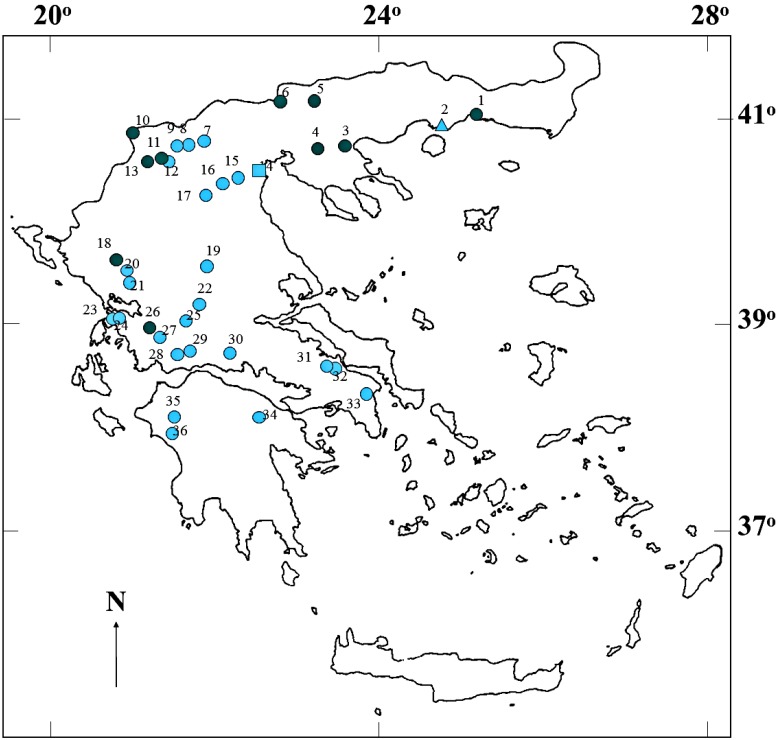
Map of Greece showing the locations of the 36 water bodies (circles: natural lakes and reservoirs, rectangle: river, triangle: lagoon), examined for the occurrence of cyanobacterial peptides. Blue-green and light blue symbols indicate water bodies with or without blooms during sampling, respectively. The numbers 1 to 36 identify the water bodies: (1) Lake Vistonis; (2) Vasova Lagoon; (3) Lake Volvi; (4) Lake Koronia; (5) Kerkini Reservoir; (6) Lake Doirani; (7) Lake Agra; (8) Lake Vegoritis; (9) Lake Petron; (10), Lake Mikri Prespa; (11) Lake Zazari; (12) Lake Cheimaditis; (13) Lake Kastoria; (14) Aliakmon River; (15) Asomaton Reservoir; (16) Sfikia Reservoir; (17) Polyphyton Reservoir; (18) Lake Pamvotis; (19) Tavropos Reservoir; (20) Louros Reservoir; (21) Pournariou Reservoir; (22) Kremaston Reservoir; (23) Lake Saltini; (24) LakeVoulkaria; (25) Lake Kastrakiou; (26) Lake Amvrakia; (27) Lake Ozeros; (28) Lake Lysimachia; (29) Lake Trichonis; (30) Mornos Reservoir; (31) Lake Yliki; (32) Lake Paralimni; (33) Marathonas Reservoir; (34) Lake Stymfalia; (35) Pinios Reservoir; and (36) Floka Reservoir.

Cyanobacteria blooms were found in 10 out of the 36 freshwater bodies surveyed (Figure 1). HPLC analysis of the water samples resulted in the identification of six different peaks in 41 out of 101 samples (HPLC-positive samples), which had the same retention time and UV absorption spectra as purified MC-LR, MC-RR, MC-YR, anabaenopeptin A, anabaenopeptin B, and anabaenopeptilide 90A, respectively. Peaks with retention times and corresponding fractions with UV spectra of [d-Asp^3^] MC-LR, [Dha^7^] MC-LR, MC-RR, [Dha^7^] MC-RR, [d-Asp^3^, Dha^7^] MC-LR, [d-Asp^3^] MC-RR, [d-Asp^3^, Dha^7^] MC-RR, and MC-LA were not found. Three additional peaks not corresponding to any of the available purified peptides were found. One peak had the same retention time and UV spectrum with MC U1 (see Section 4.2). The UV spectrum of the second peak indicated the presence of another unidentified MC, denoted U5. The UV spectrum of the third peak indicated the presence of a putative anabaenopeptin, denoted UA1.

**Figure 2:**
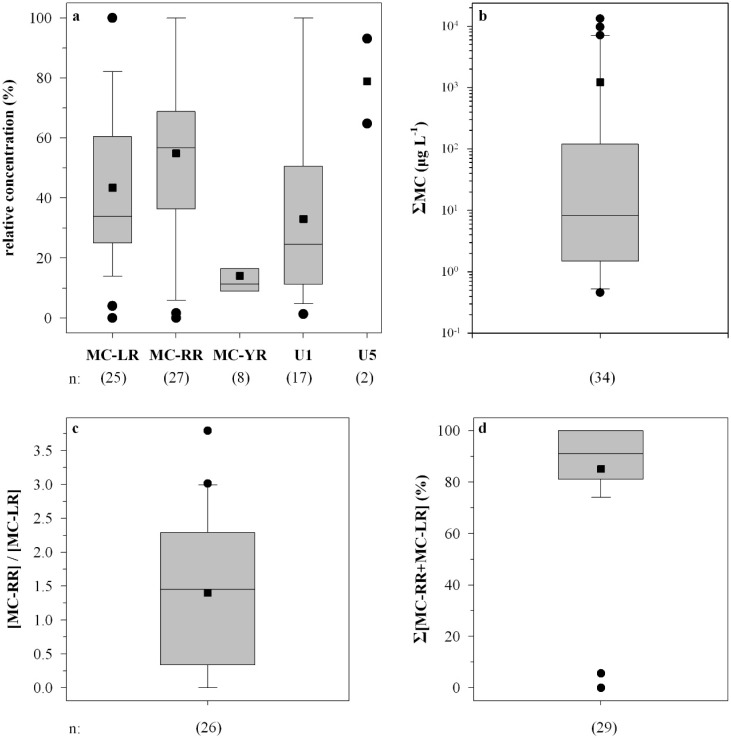
Box and whisker plots of the relative concentration of each microcystin quantified (**a**); the total microcystin concentration (ΣMC) in cyanobacterial bloom samples (**b**); the MC-RR to MC-LR intracellular concentration ratio (**c**); and the sum of MC-LR and MC-RR intracellular concentrations (%) to the ΣMC (**d**). *n* represents number of samples; U1, U5 denote the unidentified microcystin.

MC-RR and MC-LR were the predominant microcystins in 25 out of 41 HPLC-positive samples, with percentages of ΣMC ranging from 4% to 100% (mean 43%) and 2% to 100% (mean 56%), respectively (Figure 2a). MC-YR was also identified in eight samples with percentages of ΣMC ranging from 9% to 31% (mean 14%), whereas the unidentified MC U1 was predominant in 17 out of 41 HPLC-positive samples with percentages of ΣMC ranging from 1% to 100% (mean 33%). MC U5 was predominant in two bloom samples with ΣMC percentages of 65% and 93% (Figure 2a).

**Figure 3:**
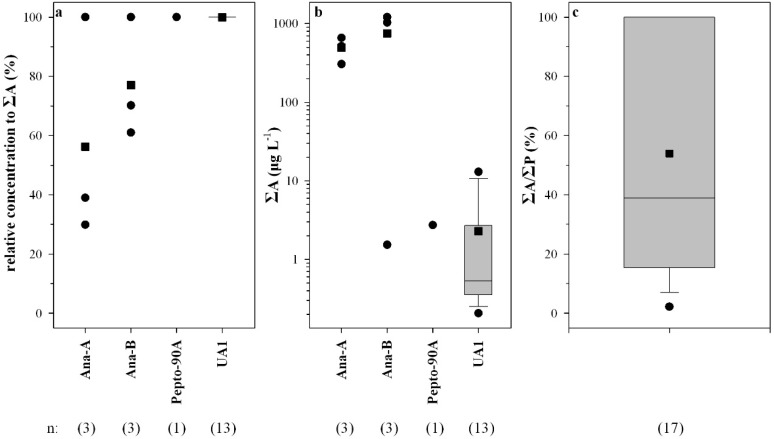
Relative concentration of each anabaenopeptin and/or anabaenopeptolide detected (**a**); total anabaenopeptin concentration (ΣΑ) (**b**); and the ratio of ΣΑ to total peptide concentration (ΣP) in water samples in which anabaenopeptins and/or anabaenopeptolides were found (**c**). UA1 denotes the unidentified putative anabaenopeptin.

Anabaenopeptin B was predominant in three samples with percentages of ΣA ranging from 61% to 100% (mean 77%), whereas anabaenopeptin A was predominant in one sample (Figure 3a). Anabaenopeptolide 90A was the only peptide found (Figure 3a) in one sample from Lake Mikri Prespa. The putative anabaenopeptin UA1 was the only anabaenopeptin found in 13 samples (Figure 3a) and the only peptide found in six out of 41 HPLC-positive samples.

### 2.2. Peptide Concentrations and Occurrence Patterns

Microcystins were found in 95% of the samples analyzed by HPLC, ELISA, and PP1IA, anabaenopeptins in 18.2% of the samples, and anabaenopeptilides in one sample (Table 1). Microcystins were found in 91% of the lakes sampled. The total intracellular microcystin concentration (ΣMC) measured in the water samples ranged from 0 to 13,230 µg·L^−1^ (Figure 2b). The MC-RR to MC-LR ratio ranged from 0.1 to 2.7 (mean 1.5) (Figure 2c). The sum of MC-LR and MC-RR percent relative concentration ranged from 70% to 100% (mean 83%), with the exception of two samples (Figure 2d). Total intracellular anabaenopeptin content (ΣA) ranged from undetectable to 1730 µg·L^−1^ (Figure 3b). ΣA constituted up to 100% of the total peptide content ΣP (Figure 3c).

**Table 1:** Number of samples per water body surveyed in this study and frequency of MC detection.

Number of Samples	Sampling Frequency		
	1	2–10	>10
total (*n*)	20	41	54
with MC (*n*)	15	39	54
frequency (%)	75	95	100

**Figure 4:**
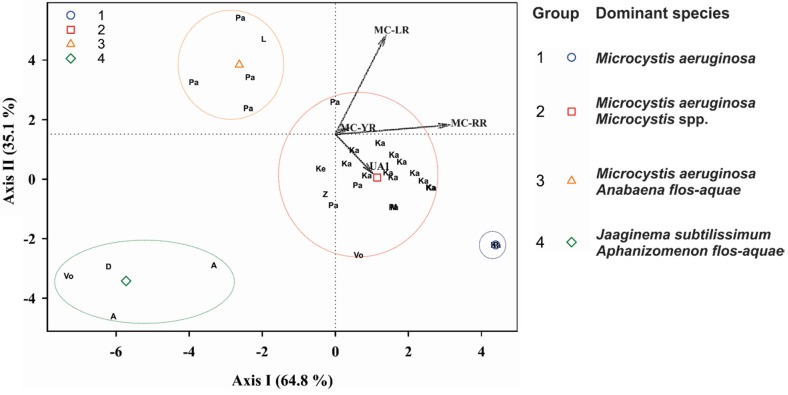
Scatter plot of the canonical scores for the two discriminant functions (Axis I and Axis II) of Discriminant Analysis and dominant cyanobacteria species for each group. Analysis of the intercorrelations between the variables: relative concentration of each peptide to the ΣMC. The variables are indicated by vectors. Points were plotted as two-digit letters, indicating freshwaters. Geometrical symbols represent the group centroids of the groups obtained by Cluster Analysis (not shown). Abbreviations: D: Doirani, Vo: Volvi, Ke: Kerkini, Μ: Mikri Prespa, Ζ: Zazari, Ka: Kastoria, P: Pamvotis, Α: Amvrakia. LR: MC-LR, RR: MC-RR, YR: MC-ΥR, UA1: putative anabaenopeptin UA1.

Cluster analysis was applied to the HPLC-positive samples, using as variables the percentage of each peptide in the total peptide concentration. The samples formed four distinct groups (cluster tree not shown). Discriminant analysis of those groups (Figure 4) revealed that most of the samples follow a pattern in microcystin dominance (Groups 1 and 2), where MC-RR and MC-LR are always the predominant microcystins, followed by MC-YR or other microcystins or anabaenopeptins. In Groups 1 and 2, the dominant species were *Microcystis aeruginosa* and other *Microcystis* spp. Two other distinct groups showed different patterns of microcystin predominance: for Group 3, where only MC-LR is predominant with percentages with respect to the ΣMC >65%; the samples were dominated by *Microcystis aeruginosa* and *Anabaena* (*Dolichospermum*) *flos-aquae*. Group 4 did not contain either MC-LR or MC-RR, and its samples were dominated by the filamentous *Jaaginema subtilissimum* and *Aphanizomenon flos-aquae* (Figure 4). A temporal and spatial variation in the concentrations of the microcystins and anabaenopeptins was observed: in Lake Mikri Prespa ΣMC was much higher in the 1999 bloom than in the 2000, whereas the contrary was observed in Lake Pamvotis (Table S2). In Lake Kastoria ΣMC differed by 2–4 orders of magnitude in different sampling stations (Table S2).

In HPLC-positive samples, ΣMC values did not differ significantly (ANOVA *p* > 0.1 for all pairs) between the three methods used (HPLC, PP1IA, ELISA). There was a high correlation in the ΣMC values between all pairs of methods (data not shown). Some HPLC-negative samples had ΣMC values >0.5 µg·L^−1^ (HPLC detection limit) when measured using ELISA or PP1IA (Table S1). In those samples ΣMC values ranged from 1 to 20 µg·L^−1^ water. In HPLC-negative samples with ΣMC values between 0.1 and 10 µg·L^−1^, there were no significant differences (ANOVA *p* > 0.1) between the values obtained with ELISA and PP1IA and there was a high correlation between the values (*r*^2^ = 0.759, *p* < 0.05). In HPLC-negative samples with ΣMC values <0.1 L^−1^, there were significant differences (ANOVA *p* < 0.05) between the values obtained with ELISA and PP1IA. The average ΣMC value obtained with ELISA was twice as high as the average ΣMC value obtained with PP1IA (data not shown).

### 2.3. Risk Assessment

**Figure 5:**
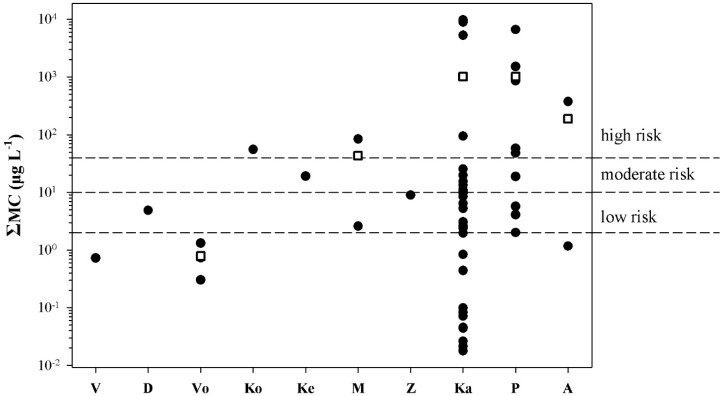
Distribution of the ΣMC values in freshwater bodies with blooms; V: Vistonis, D: Doirani, Vo: Volvi, Ko: Koronia, Ke: Kerkini, Μ: Mikri Prespa, Ζ: Zazari, Ka: Kastoria, P: Pamvotis, Α: Amvrakia. Each point represents the mean ΣMC value estimated by HPLC, PP1IA, and ELISA. Rectangles indicate mean ΣMC values.

All samples collected from water bodies with blooms, with the exception of four samples from three lakes (Lakes Vistonis, Volvi, and Amvrakia), had ΣMC values that correspond to a degree of risk when used as recreational waters (Figure 5).

**Figure 6:**
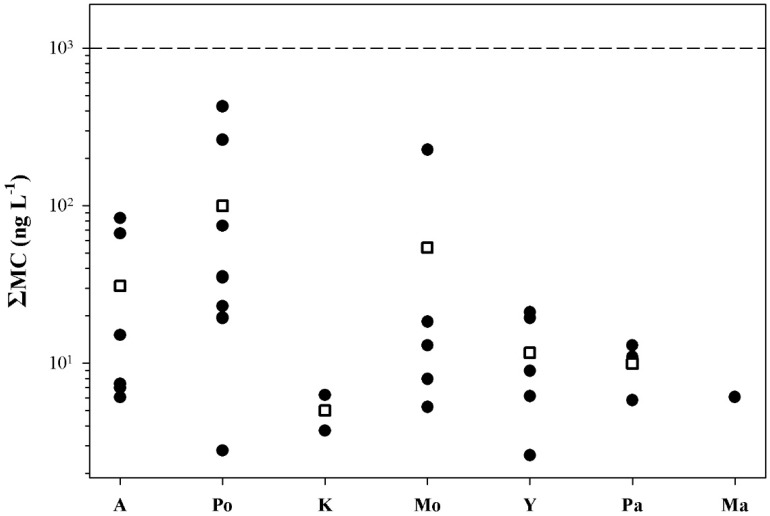
Distribution of the ΣMC values in freshwater bodies without blooms used for drinking water; A: Aliakmon, Po: Polyphyton, K: Kremaston, Mo: Mornos, Y: Yliki, Pa: Paralimni, Ma: Marathonas. Each point represents the mean value of the methods (PP1IA, ELISA) used. Rectangles indicate mean ΣMC values. The dashed line indicates WHO’s provisional guideline.

**Figure 7:**
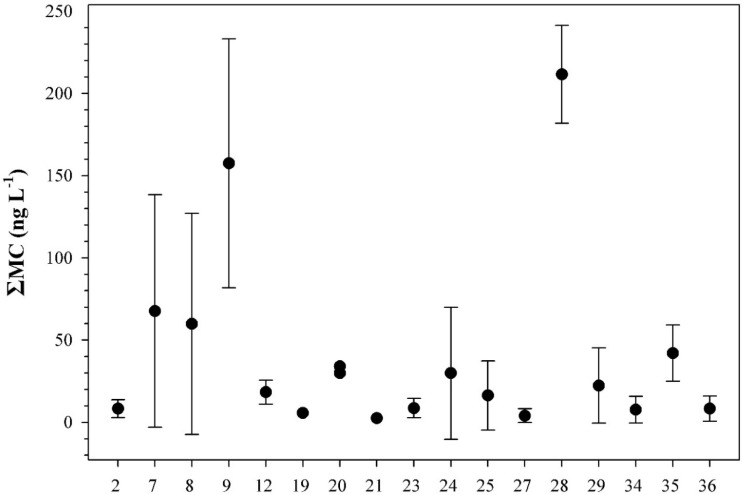
Distribution of the ΣMC values in freshwater bodies without blooms used for recreation or other purposes. Numbers on the *x*-axis indicate the freshwater number given in Figure 1. Each point represents the mean value and the standard deviation of the methods (PP1IA, ELISA) used.

In all freshwater bodies with blooms, accidental ingestion of <2 mL of water results in doses that exceed the tolerable daily intake TDI (see Table 2). In Lakes Kastoria and Pamvotis, accidental ingestion of <80 mL of bloom-containing water would result in doses over the lowest-observed-adverse-effect level (LOAEL) (see Table 2). All samples collected from freshwater bodies used as drinking water had ΣMC values below WHO’s provisional guidelines (Figure 6). All samples collected from freshwater bodies without blooms had ΣMC values below 250 ng·L^−1^ (Figure 7), and thus no risk is posed.

**Table 2:** Minimum water volume to be ingested to reach tolerable daily intake (TDI), no-observed-adverse-effect level (NOAEL), and lowest-observed-adverse-effect level (LOAEL) doses in bloom-containing water bodies of Greece.

		Water Volume (L)									
		Vistonis	Doirani	Volvi	Koronia	Kerkini	Mikri Prespa	Zazari	Kastoria	Pamvotis	Amvrakia
TDI ^a^	child	0.4	0.027	0.3	0.007	0.01	0.004	0.03	3.4 × 10^−5^	3.0 × 10^−5^	3.0 × 10^−4^
	adult	2.2	0.16	1.8	0.04	0.09	0.02	0.18	2.1 × 10^−3^	1.8 × 10^−3^	3.0 × 10^−3^
NOAEL ^b^	child	376.6	27.7	302.8	7.2	15.8	3.7	30.1	0.034	0.031	0.5
	adult	2259.8	166.0	1816.8	43.3	94.9	22.3	180.8	0.206	0.181	3.0
LOAEL ^c^	child	941.6	69.2	757	18	39.5	9.3	75.3	0.08	0.075	1.2
	adult	5649.7	415.2	4542.1	108.3	237.3	55.8	452.1	0.516	0.45	7.5

^a^ 0.04 μg·MC·kg^−1^ bw; ^b^ 40 μg·MC·kg^−1^ bw; ^c^ 100 μg·MC·kg^−1^ bw; ^d^ 10 kg bw; ^e^ 60 kg bw; for details see Section 4.3.

## 3. Discussion

This study demonstrates the widespread and persistent occurrence of microcystins in Greek water bodies, providing further information on numerous freshwater bodies not exploited before [17,19,21]. Microcystins were found in more than 90% of the samples and freshwater bodies investigated, confirming previous studies [16,17,19] that indicate that a very high percentage of the blooms in Greece are toxic. The likelihood of microcystin detection depends to some extent on the frequency of sampling [22]; microcystins in this study were found in only 75% of the waterbodies that were sampled once, but in 95% of those sampled 2–10 times and in 100% of those sampled more than 10 times. The fact that 75% of the freshwaters sampled once (most of them are oligotrophic or mesotrophic [23,24] freshwater bodies without reported blooms) contain microcystin(s) shows that microcystin-producing strains also seem to occur in lakes without blooms, as previously observed [20]. In this work we sampled lakes and reservoirs of different size, depth, altitude typology, and trophic status [23,24]; cyanobacterial blooms dominated mainly by *Microcystis* and *Anabaena* (*Dolichospermum*) were only observed in shallow, eutrophic freshwater bodies [24].

Microcystins MC-RR, MC-LR, and MC-YR were the main toxin constituents of the Greek bloom samples. The presence of these three microcystins in cyanobacterial bloom samples has been described in southern and central Europe in countries like Portugal, France, Germany, Poland, and in other parts of the world including Japan and South Korea (see [16] and references therein). In Czech reservoirs the main variants of MCs were MC-LR, MC-RR, and MC-YR, regardless of whether the bloom was dominated by *Microcystis* or other taxa [25]. Our data show that MC-RR, MC-LR, and MC-YR predominance is linked to *Microcystis*-dominated blooms. Several studies (e.g., [26,27]) indicate that the genetic composition of the bloom could impact the concentrations and congeners of the microcystins produced. Interestingly, in Lake Pamvotis MC-RR is absent, indicating that the dominant *Microcystis aeruginosa* and *Anabaena (Dolichospermum) flos-aquae* produce mainly MC-LR. The unidentified MC U1, recognized previously in Greek samples [16], was also found to be predominant in many blooms, thus more research effort is needed to be identified.

The concentrations of anabaenopeptins produced by natural populations are determined for the first time in this study. Despite the various biological activities (e.g., [10,28,29] that anabaenopeptins and related compounds exhibit, not many studies exist on their occurrence in blooms (e.g., [9,10,16,30,31]. In an anabaenopeptilide mutant *Anabaena* 90 strain, the anabaenopeptins were increased in content, possibly to compensate for the lack of anabaenopeptilide [32]. Repka *et al.* [32] suggested that anabaenopeptins and anabaenopeptilides, which both belong to the family of serine protease inhibitors, have similar functions in the cell. Furthermore, in the *Anabaena* 90 strain, decreased anabaenopeptin A and C contents were accompanied by increased anabaenopeptilide 90B contents [13]. In this study, anabaenopeptolide 90A was the only peptide found in one sample from Lake Mikri Prespa and the putative anabaenopeptin UA1 was the only peptide found in six samples in a *Microcystis wesenbergii*-dominated bloom. Similarly, in Lake Averno (Italy) anabaenopeptin B and F were found in the absence of microcystins [33]. Those findings support the suggestion by Tonk *et al.* [13] that cyanopeptolins, anabaenopeptins, and anabaenopeptilides are constitutively produced and thus, the production of cyanopeptolins, anabaenopeptins, and anabaenopeptilides resembles the constitutive production of microcystins. The evidence originating from the study of strains, however, are contradictory: none of the Portuguese microcystin-producing *Microcystis aeruginosa* strains were found to contain microginin, microviridin, anabaenopeptin, or aeruginosinamide [13]. Anabaenopeptins and aeruginosinamide were also absent in genotypes that produce microcystins [8,15]. Welker *et al.* [9] reported a high percentage of microcystin and cyanopeptolin co-production. Nonetheless, the high (>1000 µg·L^−1^) anabaenopeptin concentration we found in some samples indicates that there may be some impact, at least on the ecology and the food web structure of the aquatic ecosystems, due to the presence of anabaenopeptins and related compounds. Recently, an anabaenopeptin-producing *Microcystis flos-aquae* strain was found to strongly inhibit the growth of a freshwater amoeba, indicating that toxic bioactive compounds other than MCs are of great importance for amoebae grazing [34].

The data presented show that the HPLC, ELISA, and PP1IA methods are highly correlated, especially at high MC concentrations. HPLC may give false-negative results in the MC concentration range of 0.5 to 5 μg·L^−1^. ELISA overestimates MC concentration and may give false positive results for concentrations <0.1 μg·L^−1^. In such cases, the use of more than one method to measure MC concentrations is necessary (see [3,22] and references therein). The intracellular MC concentrations we found in 90% of the samples are similar to those reported in other countries (see [3,22]). However, the maximum intracellular MC values measured in Lakes Kastoria and Pamvotis, exceeding 10,000 μg·L^−1^, are among the highest reported in the literature, being lower than only two values reported from Algeria [35] and Germany [36]. All freshwater bodies in Greece where dense blooms occur are, by definition, above the WHO’s Guidance Level 3 [5] and in most of them intense recreational and other activities take place [23]. Recently, it was shown [18] that in Lake Pamvotis increasing water temperature and nutrient loads may act synergistically to promote cyanobacterial dominance and persistence. Our data confirm that heavy blooms producing very high microcystin concentration were already occurring in the 1990s. Thus in Lakes Kastoria, Pamvotis, Amvrakia, Zazari, and Vistonis, and Kerkini Reservoir, action should be taken in order to prevent contact with scum, possibly by prohibiting swimming and other water-contact activities, to initiate a public health investigation, and to inform the relevant authorities, according to WHO guidelines [5].

A key question for recreational exposure is whether cyanobacteria are sufficiently toxic to threaten human life. In a theoretical worst-case estimate presented by Chorus and Fastner [4], 17 mL of cyanobacterial cell material can be lethal for a small child. At the maximum concentrations of 10,000 and 13,200 μg·L^−1^ measured in Lakes Kastoria and Pamvotis, respectively, a toxic dose for an averagely sensitive 10-kg toddler would require swallowing approximately 4 L of water. Repeated exposure to scum material may occur during a holiday at a campsite or resort located beside eutrophic water bodies, if residents go swimming regularly during the summer, or via sports activities. In Lakes Kastoria and Pamvotis, accidental ingestion of <80 mL of bloom-containing water would result in doses over the LOAEL, *i.e.*, in the dose range that causes liver damage in animal studies, thus liver damage in the exposed person is possible. If humans are more sensitive than mice or pigs, or if a toddler is more sensitive than the average human being and if long-term effects are not sufficiently accounted for in the available studies, liver damage is even more likely [4]. Concerning freshwater bodies used for drinking water in Greece, the maximum concentrations were close to WHO’s guideline values [6] only in Polyphyton Reservoir and Lake Mornos. However, the presence of potentially toxic cyanobacteria that were not previously known to occur, such as *Aphanizomenon ovalisporum* in Lake Lysimachia [20], is an indication that potential problems and hazards may arise in the future through the use of the lake water.

## 4. Experimental Section

### 4.1. Sample Collection

Thirty-six lakes and reservoirs were sampled between 1996 and 2004. Geographical locations of the sampling sites are shown in Figure 1. Water samples (*n* = 104) were collected from the surface layer (0–35 cm) during the warm period of the year (May–October). In addition, 1 m and 2 m depth samples were collected from Lakes Kastoria and Pamvotis with a Nansen-type sampler. Sampling frequency differed between water bodies and ranged from biweekly to a single sampling. The water samples, with or without blooms (100–1500 mL), were kept in airtight polyethylene bottles. Bottles were placed in insulated boxes in the dark, transported to the laboratory, filtered through Whatman GF/C filters within 12 h of collection, and stored at −20 °C.

### 4.2. Peptide Extraction, Assays, and High-Performance Liquid Chromatography

Whatman GF/C filters were sliced with a sterile scalpel, placed in glass tubes with 20 mL 75% (*v*/*v*) methanol, and sonicated (Labsonic-U; Braun, Melsungen, Germany) in an ice-bath for 15 min. The resulting crude extract was stirred for 1 h at room temperature, then centrifuged at 13,000× *g* for 10 min. The supernatant was collected and the pellet re-extracted twice. The three supernatants were pooled together, dried in an air stream, and the residue was dissolved in 1 mL Milli-Q water. Half of the volume of the aqueous extract was used for ELISA and Protein Phosphatase 1 inhibition assay (PP1IA). The other half (0.5 mL) was used to form a 1 mL 50% (*v*/*v*) methanol solution that was analyzed by high-performance liquid chromatography (HPLC).

ELISA, PP1IA, and HPLC were performed according to Gkelis *et al.* [27]. HPLC-separated compounds were identified on the basis of their UV spectra and the comparison of the peak retention time with the retention time of the standards available or the retention time of the unidentified microcystins (U1–U4) and anabaenopeptin [16,20]. The qualitative standards used were MC-LR and its demethylated variants [d-Asp^3^] MC-LR, [Dha^7^] MC-LR, and [d-Asp^3^, Dha^7^] MC-LR; MC-RR and its demethylated variants [d-Asp^3^] MC-RR, [Dha^7^] MC-RR, [d-Asp^3^, Dha^7^] MC-RR, MC-LA, and MC-YR; anabaenopeptins A and B; and anabaenopeptilides 90A and 90B [11,37]. MC-LR and anabaenopeptin A were used as external standards for the quantification of MCs and anabaenopeptins/anabaenopeptilides, respectively. The identities of the peptides were verified by determining their molecular masses by HPLC-electron spray ionization mass spectrometry, using an Esquire mass spectrometer (Bruker Daltonics, Billerica Bremen, Germany) equipped with an ion trap interface coupled to an Agilent Technologies 1100 series HPLC system (for a detailed description see [38]). Quantification of unknown compounds was based on the assumption that fractions having the same λ_max_ as microcystin or anabaenopeptin/anabaenopeptilide standards are related to microcystins or anabaenopeptins/anabaenopeptilides, respectively.

Samples in which at least one peptide was identified and quantified by HPLC are referred to as HPLC-positive samples. The limit of determination and quantification was 0.3 and 0.6 μg·L^−1^ per peak, respectively, in HPLC, whereas in ELISA and PP1IA it was 0.1 and 0.15 μg·L^−1^, respectively. Results are given in ng or μg MC-LR equivalents L^−1^ water for ELISA and PP1A and in μg MC L^−1^ water for HPLC, respectively. The total intracellular microcystin concentration (ΣMC, µg·L^−1^) and the total anabaenopeptin concentration (ΣA, µg·L^−1^) in a sample are defined as the sum of the content of the individual microcystins and anabaenopeptins measured in each sample, respectively.

### 4.3. Risk Assessment

The risk from the presence of MCs in lakes was assessed by comparing the total intracellular MC concentration (ΣMC, µg·L^−1^) in water samples to the thresholds established by WHO.

According to WHO [5], in recreational waters ΣMC values in the range of 2–10 µg L^−1^ correspond to low risk, ΣMC values in the range of 10–40 µg·L^−1^ correspond to moderate risk, and ΣMC values >40 µg·L^−1^ correspond to high risk [39]. The amounts of water that have to be accidentally consumed in order to reach the tolerable daily intake (TDI; 0.04 µg·kg^−1^ body weight), no-observed-adverse-effect level (NOAEL; 40 µg·kg^−1^ body weight), and lowest-observed-adverse-effect level (LOAEL; 100 µg·kg^−1^ body weight) thresholds [40], were estimated according to the formula:

(1) $$a_{\text{w}}=\;\frac{L\;\times \;bw}{\Sigma MC}$$

where:

*α*_w_ = amount of water (L) that have to be accidentally consumed in order to reach each threshold.

*L* = threshold (TDI, NOAEL, LOAEL) value (µg·kg^−1^ body weight).

*bw* = child (10 kg) or adult (60 kg) average body weight (kg).

ΣMC = total intracellular MC concentration (µg·L^−1^).

In lakes used as drinking water supplies, risk assessment was carried out by comparing the total intracellular MC values (ΣMC, µg·L^−1^) in raw water samples, using the WHO provisional guideline (1 µg·L^−1^ water).

### 4.4. Statistical Analysis

Central tendency (average, median, and confidence intervals) and variance (range, percentiles, standard error, and standard deviation) were used for the description of variables. Statistical analyses, performed with SPSS, included cluster analysis and discriminant analysis [41]. Discriminant analysis results were compared to dominant species occurrence data published earlier [24].

## 5. Conclusions

This study showed the widespread and persistent occurrence of microcystins in Greek water bodies. The high percentage of microcystins (>90%) in the samples and freshwater bodies investigated indicates that blooms in Greece are toxic and that microcystin-producing strains also seem to occur in lakes without blooms. Anabaenopeptins and anabaenopeptolide 90A were the only peptides found in some samples, thus corroborating that some peptides are constitutively produced by bloom-forming cyanobacteria, like microcystins do. The anabaenopeptin concentration in blooms, determined for the first time in this study, can exceed 1000 µg·L^−1^, suggesting there may be some impact, at least on the ecology and the food web structure of the aquatic ecosystems. In freshwater bodies where heavy blooms occur, there is a risk posed by microcystins and action should be implemented according to World Health Organization guidelines.

## Supplementary Files

Supplementary File 1
