# Supplementary material for: Cyanobacterial Toxic and Bioactive Peptides in Freshwater Bodies of Greece: Concentrations, Occurrence Patterns, and Implications for Human Health
Source: Mar Drugs. 2015 Oct 12;13(10):6319–35. doi: 10.3390/md13106319 (PMC4626692; doi:10.3390/md13106319)
Supplement: Supplementary File 1 [file marinedrugs-13-06319-s001.docx]

Supplementary Materials

**Table S1.** Sample number, waterbody sampled, sampling date and station, and total intracellular microcystin concentration (ΣMC) measured by HPLC, PP1IA, and ELISA.

| **Sample Numer** | **Waterbody** | **Collection Date** | **Sampling Site** | **Depth (m)** | **ΣMC (μg·L^−1^)** | | |
| --- | --- | --- | --- | --- | --- | --- | --- |
|  |  |  |  |  | **HPLC** | **PP1IA** | **ELISA** |
| 1 | Vistonis | 2-8-99 | S_1_ | 0 | 0.884 | 0.225 | 1.062 |
| 2 |  |  | S_2_ | 0 | - ^a^ | 0.002 | 0.014 |
| 3 | Vasova | 2-8-99 |  | 0 | - | 0.004 | 0.012 |
| 4 | Volvi | 31-7-00 |  | 0 | - | 0.375 | 0.230 |
| 5 |  | 3-8-99 |  | 0 | 1.321 | 0.513 | 0.372 |
| 6 |  | 12-7-00 |  | 0 | 0.457 | 0.986 | 2.500 |
| 7 | Koronia | 11-9-04 |  | 0 | n.a. | n.a. | n.a. |
| 8 | Kerkini | 3-8-99 |  | 0 | 11.736 | 25.284 | 20.364 |
| 9 | Doirani | 3-8-99 |  | 0 | 14.451 | 0.066 | 0.117 |
| 10 | Agra | 5-8-99 |  | 0 | - | 0.018 | 0.118 |
| 11 | Vegoritis | 17-7-00 |  | 0 | - | 0.012 | 0.107 |
| 12 | Petron | 5-8-99 |  | 0 | - | 0.104 | 0.211 |
| 13 | Mikri Prespa | 5-8-99 |  | 0 | 107.398 | 74.072 | 70.410 |
| **14** ^b^ |  | 21-10-01 |  | 0 | - | 2.423 | 2.780 |
| 15 | Zazari | 5-8-99 |  | 0 | 8.677 | 5.010 | 13.271 |
| 16 | Cheimaditis | 5-8-99 |  | 0 | - | 0.013 | 0.023 |
| 17 | Kastoria | 1-10-00 | S_1_ | 1 | - | 0.986 | 3.793 |
| **18** |  | 3-7-00 | S_1_ | 0 | - | 1.250 | 0.423 |
| 19 |  | 26-7-99 | S_1_ | 0 | 2.614 | 1.397 | 5.000 |
| 20 |  | 20-9-00 | S_2_ | 0 | 6.626 | 15.235 | 8.430 |
| 21 |  | 1-10-00 | S_1_ | 0 | 7.960 | 4.874 | 6.457 |
| 22 |  |  | S_1_ | 1 | 0.684 | 1.113 | 4.088 |
| 23 |  | 20-9-00 | S_1_ | 0 | 13.506 | 13.599 | 19.500 |
| 24 |  | 17-7-00 | S_1_ | 0 | 1.800 | 1.644 | 4.083 |
| 25 |  | 5-8-99 |  | 0 | n.a. | 7.549 | 13.105 |
| **26** |  | 9-7-96 | S_3_ | 1 | - | 3.091 | n.a. |
| **27** |  |  | S_4_ | 1 | - | 2.636 | n.a. |
| **28** |  |  | S_2_ | 1 | - | 9.751 | n.a. |
| 29 |  | 1-8-96 | S_2_ | 1 | 44.471 | 144.266 | n.a. |
| 30 |  |  | S_3_ | 1 | 4.952 | 17.167 | n.a. |
| 31 |  | 27-8-96 | S_3_ | 1 | 9695.050 | 9915.287 | n.a. |
| 32 |  |  | S_4_ | 1 | 8.530 | 10.811 | n.a. |
| **33** |  | 4-10-96 | S_3_ | 1 | - | 25.327 | n.a. |
| 34 |  | 18-6-96 | S_2_ | 1 | 7093.475 | 11624.964 | n.a. |
| 35 |  | 30-10-96 | S_2_ | 1 | 6893.110 | 11024.600 | n.a. |
| 36 |  |  | S_4_ | 1 | 2658.064 | 7879.778 | n.a. |
| 37 |  |  | S_3_ | 1 | 4.037 | 35.435 | n.a. |
| 38 |  | 14-2-97 | S_4_ | 1 | - | 0.021 | n.a. |
| 39 |  |  | S_2_ | 1 | - | 0.018 | n.a. |
| 40 |  |  | S_3_ | 1 | - | 0.071 | n.a. |

**Table S1.** *Cont.*

| 41 |  | 11-4-97 | S_3_ | 1 | - | 0.044 | n.a. |
| --- | --- | --- | --- | --- | --- | --- | --- |
| 42 |  |  | S_2_ | 1 | - | 0.442 | n.a. |
| 43 |  |  | S_4_ | 1 | n.a. | 13.404 | n.a. |
| 44 |  | 9-5-97 | S_3_ | 1 | - | 0.046 | n.a. |
| 45 |  |  | S_2_ | 1 | - | 0.026 | n.a. |
| 46 |  |  | S_4_ | 1 | - | 0.083 | n.a. |
| **47** | Kastoria | 19-6-97 | S_3_ | 1 | - | 8.465 | n.a. |
| **48** |  |  | S_2_ | 1 | - | 5.253 | n.a. |
| 49 |  |  | S_4_ | 1 | - | 0.099 | n.a. |
| 50 | Aliakmon | 3-7-00 | S_1_ | 0 | - | 0.049 | 0.084 |
| 51 |  | 17-7-00 | S_1_ | 0 | - | 0.015 | 0.067 |
| 52 |  | 31-7-01 | S_2_ | 0 | - | 0.007 | 0.006 |
| 53 |  | 25-9-01 | S_2_ | 0 | - | 0.007 | 0.007 |
| 54 |  |  | S_3_ | 0 | - | 0.018 | 0.015 |
| 55 |  |  | S_4_ | 0 | - | 0.005 | 0.007 |
| 56 | Polyphyton | 17-7-00 | S_1_ | 0 | - | 0.145 | 0.708 |
| 57 |  |  | S_2_ | 0 | - | 0.058 | 0.091 |
| 58 |  | 3-7-00 | S_2_ | 0 | 0.545 | 0.037 | 0.204 |
| 59 |  |  | S_3_ | 0 | - | 0.015 | 0.056 |
| 60 |  | 25-9-01 | S_4_ | 0 | - | 0.025 | 0.045 |
| 61 |  |  | S_3_ | 0 | - | 0.021 | 0.017 |
| 62 |  | 31-7-01 | S_4_ | 0 | - | 0.034 | 0.012 |
| 63 |  |  | S_3_ | 0 | - | 0.031 | 0.008 |
| 64 |  | 4-11-01 | S_4_ | 0 | - | 0.002 | 0.004 |
| 65 | Pamvotis | 17-8-00 | S_4_ | 0 | 51.563 | 92.966 | 28.850 |
| 66 |  |  | S_2_ | 0 | 13231.060 | 3472.758 | 3177.000 |
| 67 |  | 5-8-00 | S_3_ | 0 | 1292.499 | 435.065 | n.a. |
| 68 |  |  | S_3_ | 1 | 7.269 | 6.015 | 3.910 |
| 69 |  | 17-8-00 | S_4_ | 0 | 1640.228 | 662.605 | 2255.000 |
| 70 |  | 5-8-00 | S_3_ | 2 | 5.380 | 3.008 | 3.875 |
| 71 |  | 17-8-00 | S_4_ | 1 | 124.112 | 8.458 | 11.275 |
| 72 |  | 22-7-99 | S_1_ | 0 | 22.960 | 24.123 | 9.394 |
| 73 |  | 5-8-00 | S_2_ | 0 | 1.736 | 0.814 | 3.453 |
| 74 | Tavropos | 21-7-99 |  | 0 | - | - | 0.006 |
| 75 | Louros | 16-8-00 | S_1_ | 0 | - | 0.011 | 0.057 |
| 76 |  |  | S_2_ | 0 | - | - | 0.002 |
| 77 | Pournariou | 22-7-99 |  | 0 | - | - | 0.002 |
| 78 | Kremaston | 21-7-99 | S_1_ | 0 | - | - | 0.006 |
| 79 |  |  | S_2_ | 0 | - | 0.004 | 0.004 |
| 80 | Saltini | 4-8-00 |  | 0 | - | 0.004 | 0.013 |
| 81 | Voulkaria | 4-8-00 |  | 0 | - | 0.001 | 0.058 |
| 82 | Kastrakiou | 21-7-99 |  | 0 | - | 0.001 | 0.031 |
| 83 | Amvrakia | 5-8-00 |  | 0 | 1.402 | 0.644 | 1.468 |
| 84 |  | 10-8-99 |  | 0 | 791.945 | 322.026 | 14.133 |
| 85 | Ozeros | 20-7-99 |  | 0 | - | 0.001 | 0.007 |

**Table S1.** *Cont.*

| 86 | Lysimachia | 20-7-99 |  | 0 | 0.904 | 0.233 | 0.191 |
| --- | --- | --- | --- | --- | --- | --- | --- |
| 87 | Trichonis | 20-7-99 |  | 0 | - | 0.006 | 0.039 |
| 88 | Mornos | 28-7-00 | S_2_ | 0 | - | 0.209 | 0.244 |
| 89 |  | 20-7-99 | S_1_ | 0 | - | - | 0.008 |
| 90 |  | 11-8-00 | S_2_ | 0 | - | 0.002 | 0.035 |
| 91 |  | 28-7-00 | S_1_ | 0 | - | 0.002 | 0.024 |
| 92 |  | 11-8-00 | S_1_ | 0 | - | 0.002 | 0.009 |
| 93 | Yliki | 27-7-00 | S_1_ | 0 | - | 0.004 | 0.008 |
| 94 |  |  | S_2_ | 0 | - | 0.006 | 0.033 |
| 95 | Yliki | 10-8-00 | S_1_ | 0 | - | 0.003 | 0.015 |
| 96 |  |  | S_2_ | 0 | - | 0.003 | 0.039 |
| 97 |  | 19-7-99 | S_1_ | 0 | - | 0.002 | 0.003 |
| 98 | Paralimni | 10-8-00 |  | 0 | - | 0.003 | 0.009 |
| 99 |  | 19-7-99 |  | 0 | - | 0.003 | 0.023 |
| 100 |  | 27-7-00 |  | 0 | - | 0.003 | 0.019 |
| 101 | Marathona | 22-9-00 |  | 0 | - | 0.002 | 0.012 |
| 102 | Stymfalia | 19-7-99 |  | 0 | - | 0.002 | 0.013 |
| 103 | Pinios | 5-10-00 |  | 0 | - | 0.030 | 0.054 |
| 104 | Floka | 5-10-00 |  | 0 | - | 0.003 | 0.014 |

^a^: under detection limit, n.a.: not analysed; ^b^ Sample number in bold indicates samples where HPLC-negative samples had ΣMC values >0.5 µg L^−1^ (HPLC detection limit) when measured using ELISA or PP1IA.

**Table S2.** Sample number, waterbody sampled, sampling date and station, peptide concentration in the HPLC-positive samples. For more details see Section 4.2.

| **Sample Numer** | **Waterbody** | **Collection Date** | **Sampling Site** | **Depth (m)** | **Peptide concentration (μg L^−1^)** | | | | | | | | | | |  |
| --- | --- | --- | --- | --- | --- | --- | --- | --- | --- | --- | --- | --- | --- | --- | --- | --- |
|  |  |  |  |  | **MC-LR** | **MC-RR** | **MC-YR** | **U1** | **U5** | **Ana-A** | **Ana-B** | | **UA** | | **Pepto 90B** |  |
| 1 | Vistonis | 2-8-99 | S_1_ | 0 | - ^a^ | - | - | 0.31 | 0.57 | - | - | | 0.83 | | - |  |
| 2 |  |  | S_2_ | 0 | - | - | - | - | - | - | - | | 0.38 | | - |  |
| 5 |  | 3-8-99 |  | 0 | - | 0.90 | - | 0.42 | - | - | - | | - | | - |  |
| 6 |  | 12-7-00 |  | 0 | - | - | - | 0.46 | - | - | - | | - | | - |  |
| 7 | Koronia | 11-9-04 |  | 0 | n.a. | n.a. | n.a. | n.a. | n.a. | n.a. | n.a. | | n.a. | | n.a. |  |
| 8 | Kerkini | 3-8-99 |  | 0 | 4.21 | 5.27 | - | 2.26 | - | - | - | | - | | - |  |
| 9 | Doirani | 3-8-99 |  | 0 | - | - | 4.42 | 10.03 | - | - | - | | - | | - |  |
| 13 | Mikri Prespa | 5-8-99 |  | 0 | 25.00 | 64.33 | 12.08 | 6.00 | - | - | - | | 12.96 | | - |  |
| 14 |  | 21-10-01 |  | 0 | - | - | - | - | - | - | - | | - | | 2.72 |  |
| 15 | Zazari | 5-8-99 |  | 0 | 2.20 | 4.35 | - | 2.13 | - | - | - | | - | | - |  |
| 17 | Kastoria | 1-10-00 | S_1_ | 1 | - | - | - | - | - | - | - | | 3.35 | | - |  |
| 18 |  | 3-7-00 | S_1_ | 0 | - | - | - | - | - | - | - | | - | | - |  |
| 19 |  | 26-7-99 | S_1_ | 0 | 0.65 | 1.96 | - | - | - | - | - | | - | | - |  |
| 20 |  | 20-9-00 | S_1_ | 0 | 2.40 | 4.22 | - | - | - | - | - | | - | | - |  |
| 21 |  | 1-10-00 | S_1_ | 0 | 2.95 | 4.01 | - | 0.99 | - | - | - | | - | | - |  |
| 22 |  |  | S_1_ | 1 | - | 0.68 | - | - | - | - | - | | - | | - |  |
| 23 |  | 20-9-00 | S_1_ | 0 | 5.31 | 8.20 | - | - | - | - | - | | - | | - |  |
| 24 |  | 17-7-00 | S_1_ | 0 | - | 1.80 | - | - | - | - | - | | - | | - |  |
| 29 |  | 1-8-96 | S_2_ | 1 | 13.93 | 30.55 | - | - | - | - | - | | - | | - |  |
| 30 |  |  | S_3_ | 1 | 1.39 | 3.57 | - | - | - | - | - | | - | | - |  |
| 31 |  | 27-8-96 | S_4_ | 1 | 2860.53 | 5970.06 | 864.47 | - | - | - | - | | - | | - |  |
| 32 |  |  | S_4_ | 1 | 2.14 | 6.39 | - | - | - | - | - | | - | | - |  |
| 33 |  | 4-10-96 | S_3_ | 1 | - | - | - | - | - | - | - | | - | | - |  |
| 34 |  | 18-6-96 | S_2_ | 1 | 2228.92 | 4229.97 | 634.60 | - | - | - | | - | - | - | | |

**Table S2.** *Cont*.

| 35 |  | 30-10-96 | S_2_ | 1 | 3626.89 | 2473.20 | 793.02 | - | - | 514.95 | 1209.94 | - | - |
| --- | --- | --- | --- | --- | --- | --- | --- | --- | --- | --- | --- | --- | --- |
| 36 |  |  | S_4_ | 1 | 1389.54 | 825.42 | 443.11 | - | - | 657.69 | 1029.40 | - | - |
| 37 |  |  | S_3_ | 1 | 2.42 | 1.62 | - | - | - | - | 1.53 | - | - |
| 51 | Aliakmon | 17-7-00 | S_1_ | 0 | - | - | - | - | - | - | - | 0.36 | - |
| 57 |  |  | S_2_ | 0 | - | 0.55 | - | - | - | - | - | - | - |
| 58 |  | 3-7-00 | S_2_ | 0 | - | - | - | - | - | - | - | - | - |
| 65 | Pamvotis | 17-8-00 | S_4_ | 0 | 34.92 | 3.28 | 8.20 | 5.17 | - | - | - | 7.19 | - |
| 66 |  |  | S_2_ | 0 | 13231.06 | - | - | - | - | 304.44 | - | - | - |
| 67 |  | 5-8-00 | S_3_ | 0 | 964.06 | 168.67 | - | 159.77 | - | - | - | - | - |
| 68 |  |  | S_3_ | 1 | 5.39 | - | - | 1.87 | - | - | - | - | - |
| 69 |  | 17-8-00 | S_4_ | 0 | 339.32 | 882.60 | - | 418.31 | - | - | - | - | - |
| 70 |  | 5-8-00 | S_3_ | 2 | 0.98 | 3.71 | - | 0.69 | - | - | - |  |  |
| 71 |  | 17-8-00 | S_4_ | 1 | 5.04 | 1.99 | - | 1.61 | 115.46 | - | - | - | - |
| 72 |  | 22-7-99 | S_1_ | 0 | 7.34 | 11.39 | 2.02 | 2.21 | - | - | - | 2.06 | - |
| 73 |  | 5-8-00 | S_2_ | 0 | 1.08 | 0.66 | - | - | - | - | - | - | - |
| 83 | Amvrakia | 5-8-00 |  | 0 | - | - | - | 1.40 | - | - | - | 0.63 | - |
| 84 |  | 10-8-99 |  | 0 | - | 269.08 | - | 522.87 | - | - | - | - | - |
| 85 | Ozeros | 20-7-99 |  | 0 | - | - | - | - | - | - | - | - | - |
| 86 | Lysimachia | 20-7-99 |  | 0 | 0.90 | - | - | - | - | - | - | 0.21 | - |
| 88 | Mornos | 28-7-00 | S_2_ | 0 | - | - | - | - | - | - | - | 0.32 | - |
| 89 |  | 20-7-99 | S_1_ | 0 | - | - | - | - | - | - | - | 0.50 | - |
| 90 |  | 11-8-00 | S_2_ | 0 | - | - | - | - | - | - | - | 0.35 | - |
| 100 |  | 27-7-00 |  | 0 | - | - | - | - | - | - | - | 0.53 | - |

^a^: under detection limit, n.a.: not analysed.

© 2015 by the authors; licensee MDPI, Basel, Switzerland. This article is an open access article distributed under the terms and conditions of the Creative Commons Attribution license (http://creativecommons.org/licenses/by/4.0/).
